# Spreading of SARS-CoV-2 among adult asylum seekers in refugee community shelters in Lübeck, Germany between 2020 and 2022: a mixed-cohort observational and repeated cross-sectional study

**DOI:** 10.1186/s12889-025-22120-9

**Published:** 2025-04-07

**Authors:** Daniel Alvarez-Fischer, Max Borsche, Alexander Balck, Bandik Föh, Arnim Hoischen, Fawad Hotak, Jan Reinhardt, Susanne A. Elsner, Elke Peters, Andrea Rieck, Emily L. Martin, Inga Künsting, Marc Ehlers, Alexander Mischnik, Stefan Taube, Nadja Käding, Jan Rupp, Alexander Katalinic, Christine Klein

**Affiliations:** 1 Institute of Neurogenetics, University of Lübeck, Ratzeburger Allee 160, 23538 Lübeck, Germany; 2 Institute of Systems Motor Science, Center for Brain, Behavior, and Metabolism, Lübeck, Germany; 3 Department of Medicine, University Hospital Schleswig-Holstein, Campus Lübeck, Lübeck, Germany; 4 Institute of Nutritional Medicine, University of Lübeck, Lübeck, Germany; 5 Department of Psychiatry and Psychotherapy, University of Lübeck, Lübeck, Germany; 6 Institute of Social Medicine and Epidemiology, University of Lübeck, Lübeck, Germany; 7 Health Protection Authority, City of Lübeck, Lübeck, Germany; 8 Institute of Virology and Cell Biology, University of Lübeck, Lübeck, Germany; 9 Department of Infectious Diseases and Microbiology, University Hospital Schleswig-Holstein, Campus Lübeck, Lübeck, Germany

**Keywords:** SARS-CoV-2, COVID-19, Refugees, Community shelters

## Abstract

**Introduction:**

Housing and access to healthcare pose particular challenges to asylum seekers and refugees. The main study aim was to assess their frequency of SARS-CoV-2 infection during the COVID-19 pandemic.

**Methods:**

We provide a prospective study on SARS-CoV-2 cases among adult asylum seekers/refugees in Europe over 18 months. Acute SARS-CoV-2 infection and antibody titers were determined in adult refugees living in shared accommodation in Lübeck, Germany, in fall 2020 (TP1) and spring 2021 (TP2) and compared to the results from a local population-based cohort. In spring 2022 (TP3), we determined antibody titers two years into the pandemic and one year of access to vaccination. At TP3, we additionally included a third cohort of recently arrived refugees from Ukraine.

**Results:**

At all three time points, we detected a marked increase in the infection frequency in refugee community shelters compared to the control group. Age, sex, or facility equipment did not impact the results. Refugees living with their own children in the shelter were significantly more often infected than those without. None of the PCR-positive refugees at TP1 and TP2 were aware of their infection. One year later, awareness of having had an infection was still much lower among the refugees compared to the control cohort. Only 32.9% of the asylum seekers were willing to be vaccinated compared to 85.5% in the control population at the beginning of the vaccination period. However, over 90% of the same population was vaccinated one year later. Among newly arrived refugees from Ukraine, uncertainty towards vaccination was significantly increased compared to the control cohort and the group of residing refugees.

**Conclusion:**

Refugees residing in shared accommodations represent a vulnerable group for SARS-CoV-2 infection and transmission. This increased vulnerability does not diminish over time. Initial doubts regarding vaccination are higher among refugees. While this reservation can be overcome, awareness work is paramount and has to start anew with any new refugee wave.

**Supplementary Information:**

The online version contains supplementary material available at 10.1186/s12889-025-22120-9.

## Introduction

Refugees and asylum seekers are typically confronted with particular challenges regarding housing and access to the health care system, making them a potentially highly vulnerable group during the COVID-19 pandemic [1–3]. Accordingly, viewpoints on individuals currently on the move or stranded in primary camps at the European Union border have been published early in the pandemic [4–6]. In general, refugees are at increased risk of suffering from a plethora of partly rare pulmonary infections [7] and might be excluded from access to healthcare services in Europe [8]. Mass accommodation in community shelters, as currently practiced, for example, in Germany, might be disadvantageous in many ways, not only in the context of transmittable diseases [9]. Moreover, the living situation of refugees cannot be considered homogenous and represents a complex topic influenced by, amongst others, structural and political factors, the type of journey, as well as the country of origin and destination [10].

Thus far, refugees who already arrived in their country of destination have not yet been the focus of any scientific studies on the Covid-19 pandemic, and prospective longitudinal studies of the spread of SARS-CoV-2 in these individuals in Europe have not been performed to date. A cross-sectional study among homeless men living in mass accommodation in France showed high positivity rates for SARS-CoV-2 infection (8.4%) at the beginning of the pandemic [11, 12]. Several characteristics render asylum seekers more susceptible to contracting respiratory diseases such as COVID-19 [13, 14]: Most (62%) live in community shelters or multigenerational inner-city housing, where social distancing is often difficult. Early detection of cases is challenging due to restricted access to national healthcare plans and healthcare services [13, 14]. Public health campaigns do not reach this group sufficiently, and it is difficult for them to be included n population-based studies [3, 14, 15].

The main goal of the study was to investigate in a longitudinal, prospective manner SARS-CoV-2 infection rates by PCR- and seroconversion among adult refugees in Lübeck, a city of approximately 220,000 inhabitants in Northern Germany. We hypothesized that the frequency of SARS-CoV-2 infection is higher among refugees in community shelters compared to the general population. Therefore, the number of infected among adult refugees was compared to a large local prospective population-based cohort from the “ELISA” (Lüb**e**ck **L**ongitudinal **I**nvestigation of **SA**RS-CoV-2 Infection-study), a two-year, population-based longitudinal investigation of SARS-CoV-2 prevalence and recovery in the Lübeck area [16, 17]. Thereby, we aimed to elucidate whether the increased infection frequency diminishes over time compared to a local control cohort. Furthermore, we aimed to identify factors mediating this increased infection frequency of SARS-CoV-2 infections among refugees in community shelters with special interest in the awareness of being infected with SARS-CoV-2 compared to the local control cohort. Finally, as vaccination hesitancy seems to be a characteristic problem in Germany or German-speaking countries, we aimed to elucidate the attitude towards vaccination and its development among both cohorts.

## Methods

### Study population

In the spring and summer of 2020, at the beginning of the COVID-19 pandemic, the Lübeck area was a low-prevalence region, but SARS-CoV-2 infection rates increased markedly from October 2020 to May 2022 [16]. Asylum seekers residing in community shelters were included in this study at three time points. The first investigation occurred from November 23rd to December 3rd, 2020 (time point 1, TP1), and the second one from February 17th to February 24th, 2021 (time point 2, TP2). The third evaluation was performed from May 11th to May 25th (time point 3, TP3). We contacted the facilities and included them in the study planning. One week before the start of the investigation, the study team personally visited the facilities and informed all residents about the study in a door-to-door approach. We explained that the study was anonymous and that there would be no disadvantage in case of non-participation. We provided study information material if there was a willingness to participate in principle. All participants who declared potential willingness ended up participating in TP1. All contact with the subjects always took place in the presence of an interpreter and a trusted person of the institution. Any adult who declared interest in participation was included. The two study exclusion criteria were age below 18 years and inability to give informed consent. We had no information about the reason why we could not reach an eligible person or why somebody refused to participate. One year later, in May 2022, we returned to the same shelters to re-evaluate the people living there. At that time, Europe was experiencing a new wave of refugees due to the Russian invasion of Ukraine. Thus, we included recently arrived refugees from Ukraine in the follow-up evaluation and added them as a third study cohort. Further information about recruitment and the study population is provided in the additional file 1.

### SARS-CoV-2 testing

At TP1 and TP2, trained study personnel performed deep nasal and oropharyngeal swabs, analyzed by quantitative Real-Time PCR as previously described [16, 17]. Moreover, venous blood samples were used for antibody testing using the EUROIMMUN SARS-CoV-2 S1 IgG (#EI 2606-9601-2 G) ELISA according to the manufacturer’s instructions. At TP3, no PCR testing was performed. Due to vaccination, which became available for most of the cohorts between TP2 and TP3, S1 antibody levels were no longer suitable for detecting infection. Thus, we defined NCP antibody levels > 1.1 as seropositive, indicating a passed SARS-CoV-2 infection at TP3. Here, antibody levels were determined by capillary blood taking and antibody analysis from dried blot spots as described [18, 19]. According to the manufacturer’s information, the sensitivity of the swap testing is 98.2%, and the specificity is 100%. The sensitivity of the blood drop test is 94.6%, and the specificity is 99.8%. More detailed information about the testing procedure and technical specifications is available in the additional file 2.

**Fig. 1:**
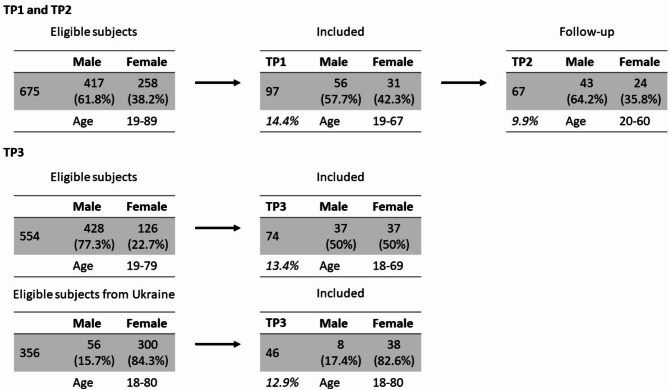
The figure summarizes the number of eligible and included subjects

Results were compared to analyses from the population-based ELISA study conducted in the same area [16, 18]. Specifically, results of refugees residing in community shelters cross-sectionally with the population-based sample were compared to a simultaneous investigation of the ELISA study (ELISA-TP1: November 16th to December 5th, 2021; ELISA-TP2: February 1st to February 27th, 2021, ELISA-TP3: April 1st to May 25th). In the community shelters, there was no general testing strategy at any of the evaluation time points. The number of included participants at the corresponding time points is depicted in Fig. 1. One resident developed symptoms six weeks before TP2. The resident was tested, and a SARS-CoV-2 infection was confirmed and reported to the authorities, who decided to test all residents of the largest facility regardless of disease symptoms or contact tracing. This testing was performed independently of the study.

### Demographics, main outcomes, and potential effect modifiers

The main outcome was acute or past SARS-CoV-2 infection, according to PCR and antibody results. Regarding demographics and potential influencing factors, assessed via questionnaires, we analyzed data on age, sex, country of origin, living with own children in the shelter, awareness of infection, and attitudes towards vaccination.

### Statistics

The frequency of virus- and antibody positivity among the study group was compared to the population-based ELISA cohort at corresponding time points [16, 18] using the χ^2^ test. Data are given as absolute numbers and percentages of the sample. We provide p-values as well as 95% confidence intervals (CI). Due to a lack of information about the distribution and other characteristics of the single houses, the selection of the sample group was not representative, and we cannot conclude a selection bias. At TP1, we included 97 individuals out of 675 eligible persons. This number leads to a margin of error of 10% with a confidence interval of 95%. The final number of included persons was higher due to the inclusion of a further group at TP3 (Fig. 1). Data were analyzed using SPSS, Jamovi, and Excel.

### Ethics

The ethics committee of the University of Lübeck approved the study (Az. 20–150). All participants gave written informed consent. All study materials were translated into the refugees’ native or official languages in their countries of origin (English, Farsi/Dari, Arabic, and Russian), and interpreters were available onsite at the study center.

### Patient and public involvement

We included local health authorities, caregivers, facility providers, and facility managers in the study’s design, conduct, reporting, and dissemination. They all agreed to support the study. The study protocol of the main ELISA study [16, 17] was already published. Thus, we wanted to follow the same protocol. The local health authorities, the caregivers, the facility providers, and the facility managers were also informed about the study results in advance. Upon completion of the study, a press release was issued for the local press. The participants were not included in the planning phase due to organizational issues.

### Data Availability

The data underlying this study are available from the corresponding author upon reasonable request.

## Results

### Study population

**Fig. 2:**
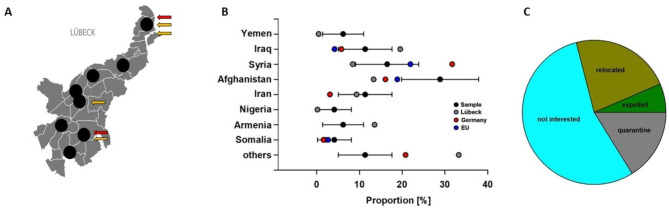
Characteristics of the study sample. **(A)** We investigated 97 asylum seekers living in community shelters distributed throughout the city of Lübeck. Arrows indicate positive PCR findings of SARS-CoV-2 (red) or positive anti-S1 IgG antibodies (orange) in each facility at time point 1 (TP1). **(B)** The bar graph shows the countries of origin of study participants with 95%-CI in black in relation to Lübeck (grey, Germany (red), and Europe. Omitted dots due to unavailable data. **(C)** At time point 2 (TP2), 67 of 97 refugees from TP1 were re-analyzed. The pie diagram depicts the reasons for drop-outs

In Lübeck, 1127 refugees lived in shared accommodation at TP1 and TP2, 675 of whom were over 18 years of age. They are spread across ten homes in the city. Out of all refugees meeting the inclusion criteria, 97 refugees (14.4%) from 15 countries residing in 8/10 community shelters (Fig. 2A) with a mean age of 37.7 years (range 19–67, standard deviation 11.7 years) (Fig. 1) were willing to participate. Since the different facilities accommodate different numbers of people, some facilities also had more study participants. In the study group, 57.9% of the participants were male and 42.1% female, corresponding to 61.8% of male and 38.2% of female refugees older than 18 years living in Lübeck at this time. The distribution of the countries of origin was representative of that of the refugees in Lübeck (Fig. 2B). Residential facilities included in the study differed in terms of the number of residents (from *n* = 52–243 per shelter) and the number of non-family members who have to share sanitary facilities and kitchens, from 1 to 8 families per facility. The ratio of healthcare providers to refugees was 1:40 in all shelters. The shelters did not differ regarding access to the health care system.

At the second time point (TP2), 67/97 refugees (69%) were available for the follow-up investigation. Reasons for drop-outs were “not interested” (56.7%), relocated without a known destination (23.3%), quarantine (16.7%), and expelled (6.7%) (Fig. 2C).

A mass outbreak was registered between TP1 and TP2 in one of the participating community shelters. Out of 129 residents, 51 were confirmed PCR-positive and one staff member was confirmed PCR-positive (data provided by the health authorities of Lübeck).

The control population has been described previously [16, 18]. In brief, 44% of participants were male with a mean age of 45.6 years (range: 18–79 years, standard deviation 15.2 years) [16].

At TP3, we re-evaluated 74 refugees and included an additional 46 refugees from Ukraine who arrived within 2–8 weeks before evaluation. At that time point (TP3), 356 refugees from Ukraine older than 18 years were registered in Lübeck (Fig. 1). Thus, we included 12.9% of the total at that time. Due to the prohibition of leaving Ukraine for men between 18 and 60 years of age, we found a strong disbalance in favor of the female sex in the Ukraine refugee population (17.4% male vs. 82.6% female). The mean age was 35.5 years (range: 18–80, standard deviation 15.1 years). We do not have missing data for the reported variables of interest. Please note the results on ten caregivers in the additional file 3.

### Infection frequency

**Fig. 3:**
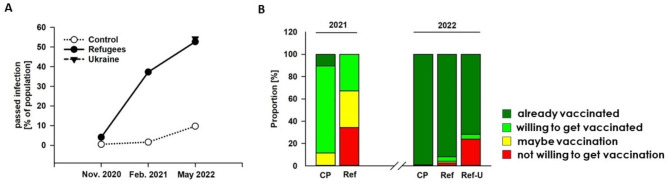
Infection frequency and attitudes towards vaccination. **(A)** We monitored the infection frequency in the refugee population and compared the results with a population-based sample of the same city. In spring 2022, we included a third group of recently arrived refugees from Ukraine. In November 2020 and February 2021, seropositivity was determined. **(B)** We surveyed attitudes towards vaccination. Compared to the control population (CP) of Lübeck, vaccination readiness was profoundly lower among the group of refugees (Ref) at TP2. However, after one year, the vast majority of refugees were vaccinated in the same refugee group. Yet, the refugees from Ukraine (Ref-U) had a more negative attitude towards vaccination than the other groups. Dark green = vaccinated, green = willing to get a vaccination, yellow = maybe vaccination, red = not willing to get a vaccination

The frequency at TP1 (November/December 2020, two months before the mass outbreak in one shelter) of both PCR-confirmed acute and passed infection (indicated by antibody positivity) was low but markedly increased compared to the population-based cohort from Lübeck (Fig. 3A). While among the asylum seekers 2/97 PCR-positive (2.1%, 95% confidence interval (CI) 0.4 to 6.3%) and 4/97 (4.1%, CI 1.4 to 9.2%) seropositive individuals were found, only 3/2547 PCR-positive individuals (0.1%, CI 0.0 to 0.3%) and 12/2547 seropositive individuals (0.5%, CI 0.3 to 0.8%) were detected in population-based control samples at the same time point, TP1 (Table 1). All individual refugees who tested positive in our study were asymptomatic and unaware of the infection. At the second timepoint, again, both acute infection and – after the mass outbreak in one shelter – seropositivity significantly increased compared to the control group (Table 1). We found in the study population 2/67 (3.0%, CI 0.5 to 9.1) PCR- and 25/67 (37.3%, CI 27.4 to 48.1%) seropositive cases compared to 2/2357 (0.1%, CI 0.0 to 0.3%) and 38/2357 (1.6%, CI 1.2 to 2.1%) in the Lübeck control population (Fig. 3A). Of note, this was also true for antibody results when the samples from the community shelter with the mass outbreak were excluded (7/32 (21.9%, CI 10.7 to 37.2%; Table 1, TP2* [results without participants from that respective shelter at the second time point]). One year later (TP3), the seropositivity for a passed infection indicated by antibodies against the nucleocapsid protein (NCP) was profoundly increased, comparing the population of refugees with the control group: 39/74 (52.7%, CI 41.3 to 62.8%) in the study population compared to 180/1849 (9.7%, CI 8.4 to 15.5%) in the control group. In the Ukrainian population, 25/46 revealed seropositivity against NCP (54.3%, CI 40.0 to 64.1%) (Fig. 3A). At no time point, age, sex, or country of origin impacted infection frequency.

**Table 1:** Distribution of SARS-CoV 2 in the study sample and the general population

Time-point	Refugees	Population-based control group [16]		Refugees	Population-based control-group [16]	
	PCR positivity		*p*-value	Seropositivity		*p*-value
TP1	2/97 (2.1%), (CI 0.4–6.3%)	3/2547 (0.1%) (CI 0-0.3%)	< 0.001	4/97 (4.1%) (CI 1.4–9.2%)	12/2547 (0.5%) (CI 0.3–0.8%)	< 0.001
TP2	2/67 (3%), (CI 0.5–9.1%)	2/2371 (0.1%) (CI 0-0.3%)	< 0.001	25/67 (37.3%) (CI 27.4–48.1%)	38/2371 (1.6%) (CI 1.2–2.1%)	< 0.001
TP2*	0/32 (0%) (CI 0-8.9%)	2/2371 (0.1%) (CI 0-0.3%)	n.a.	7/32 (21.9%) (CI 10.7–37.2%)	38/2371 (1.6%) (CI 1.2–2.1%)	< 0.001
TP3	n.a.	n.a.	n.a.	64/120 (53.3%) (CI 44.4–62.2%)	180/1849 (9.7%) (CI 8.4–11.1%)	< 0.001

The table shows the study’s results comparing refugees with cross-sectional analyses of the local population of Lübeck at the respective period at all time points (TP1 November/December 2020; TP2 February 2021; TP3 May 2022). Data are given as an absolute number, percentage, and the 95% confidence interval (CI). As in one community shelter, a mass outbreak was registered between TP1 and TP2, we show the results without participants from that respective shelter (TP2*). Chi-square tests revealed significant differences at TP1 (PCR-positivity: *p* < 0.001; Antibody-positivity: *p* < 0.026) and TP2 (PCR-positivity: *p* < 0.001; antibody-positivity: *p* < 0.001). Regarding antibody positivity at TP2, differences regarding antibody positivity were also significant if the participants from the mass-outbreak shelter were excluded (TP2*). At TP3, only NCP positivity is shown, as the vast majority of both populations were vaccinated at TP3. The results from all refugees were given, including the Ukrainian population. CI – confidence interval

“Living with own children in the shelter” was significantly positively associated with being infected at TP1 and TP2 (*p* < 0.01, Table 2). This association was even more pronounced when analyzing the home with the mass outbreak alone (*p* < 0.001, Table 2). At TP3, this was not evaluated. Almost all Ukrainians were accompanied by their children, which could have influenced the results.

**Table 2:** Relation of SARS-CoV-2 infection and living with children

	Positive	Negative	Sum	
With own children	19 (17)	30 (8)	49 (25)	
Without own children	6 (2)	42 (22)	48 (24)	
Sum	25 (19)	72 (30)	97 (49)	*p* < 0.01 (*p* < 0.001)

The table shows the impact of living with own children in a community shelter on the frequency of developing seropositivity at one of the time points. In parentheses, the shelter with the mass outbreak alone is depicted. Data are given as absolute numbers; statistical analysis was performed by calculating a Chi-square test

### Awareness of being infected

Already between the first two time points, both the prevalence and incidence of infection were markedly increased in the refugee group. As none of the individuals who tested PCR-positive in our study were aware of an acute infection, we wondered whether, after one year, awareness of a passed infection was still altered in contrast to the control population. Therefore, we asked whether each subject remembered a SARS-CoV-2 infection during the pandemic. Although we are aware that the NCP antibody titer could be below the detection threshold even after infection, we still counted NCP positivity as a passed infection. Notably, this might underestimate our result as the infection frequency was higher in the refugee population. Whereas in the control population, 34 out of 180 NCP seropositive subjects did not remember an acute infection (18.9%, CI 13.1 to 24.6%), the proportion among refugees was with 49/64 (76.6%, CI 66.2 to 86.9%) significantly higher (chi-square statistic with Yates correction is 67.421, p-value < 0.00001). In contrast, 8/23 (34.8%, CI 15.3 to 54.2) who remember an infection in the study group were negative for NCP antibodies compared to 3/149 (2.0%, CI 0 to 4.3%) in the control group (Table 3).

**Table 3:** Awareness towards infection

Time-point	Refugees	Population-based control group[16]		Refugees	Population-based control-group[16]	
	Not remembering infection despite NCP-AB positivity		*p*-value	Remembering infection despite NCP-AB negativity		*p*-value
TP3	49/74 (76.6%), (CI 66-86.9%)	34/180 (18.9%) (CI 13-24.6%)	< 0.001	8/23 (34.8%) (CI 15-54.2%)	3/149 (2.0%) (CI 0-4.3%)	< 0.001

The table shows the seropositivity of anti-spike antibodies within the study population. Note that all subjects without antibodies were from Ukraine. One of the subjects vaccinated with Pfizer and showing no seropositivity was vaccinated only once, the other only twice. CoronaVac was vaccinated twice in all of the seven subjects. Chi-square tests revealed significant differences: *p* < 0.001

### Attitudes towards vaccination

At the second time point in February 2021, when the first vaccines became available in Europe, we asked about attitudes towards vaccination. Only one-third (22/67, 32.9% (95% CI 31.1 to 34.6%) of the study population were willing to be vaccinated at this time point. Due to the vaccination priority plan (prioritizing the elderly, particularly vulnerable people, and medical personnel at the time), none of the refugees was vaccinated and had had the opportunity to get a vaccination until then. In the control cohort, 88.5% (1726/1951, of whom 202 were already vaccinated, 95% CI 87–89%) declared wanting to be vaccinated “in any case”) (Fig. 3B). One year later, however, 91.9% (68/74, CI 85.7 to 98.1%) of the refugees in the same group were vaccinated, and a further 4% wanted to be vaccinated. Only 2.7% (CI 0 to 6.4%) refused to be vaccinated (Fig. 3B). The attitudes towards vaccination among refugees were more cautious than in the ELISA control cohort, in which only 16/1849 (0.9%, CI 0.4 to 1.3%) were not vaccinated and refused vaccination. Still, the rate of about 92% vaccinated people among refugees was slightly higher than indicated in the public registries for the total population of the region of Schleswig-Holstein for this age group, namely 85.1%. Next, we found a pronounced difference in the vaccination rate and willingness to get vaccinated in the study group compared to the results from the refugees from Ukraine who have recently fled their country. While only 33/46 (71.7%, CI 58.7 to 84.8%) of the Ukrainian refugees had already been vaccinated and another 2/46 (4.4%) wanted to be vaccinated, 11/46, i.e., 23.9% (CI 11.6 to 36.2%), refused to be vaccinated, compared to 2.7% of the refugees in the first group.

## Discussion

We hereby report on a prospective observational study focusing on refugees residing in community shelters and exploring the of being infected with SARS-CoV-2 over 18 months in two different refugee cohorts, including Ukrainians. We provide robust evidence that refugees residing in shared accommodations represent a highly vulnerable group for SARS-CoV-2 infection and transmission. The increased infection frequency does not approach the infection frequency of the control group over time. With the increasing prevalence of SARS-CoV-2 in the study area, infection was associated with the presence of children in the residence, possibly due to infections in schools/daycare centers with an unnoticed spread from children and/or because having children complicates and reduces social distancing.

None of the individuals who tested PCR-positive in our study were aware of an acute infection. The rate of remembering a past infection one year later was lower than in the control cohort. This recall bias aligns with experiences from other communicable diseases [20], even though public awareness was higher compared to other infectious conditions.

We can only hypothesize why the probands did not notice disease symptoms or link them to a possible infection. One possible explanation could be that asylum seekers and refugees are typically confronted with administrative challenges to access the healthcare system [1, 14] and have to struggle with problems other than healthcare [14]. Another possible explanation might be the younger age of infected people in this overall young cohort, which might be a reason for the presence of asymptomatic infections or infections with mild symptoms [1]. Of note is that the awareness of infection seems to be reduced among refugees living in shared accommodations over the entire period of observation, underlining that it is not a transient phenomenon but rather a structural problem. Consequently, the protection of others is decreased in refugee community shelters as this is coupled with awareness. Thus, in comparable pandemic situations, event-free testing might be essential for preventing the spreading of infection in such vulnerable groups.

As vaccination hesitancy seems to be a characteristic problem of German-speaking countries, we were surprised by the lack of readiness to get vaccinated within the study group at the beginning of the vaccination period. Publications from similar subgroups in France, the US, and the UK at that time supported our finding [1, 16, 21]. We did not further explore the reasons for the lack of vaccination readiness. However, we hypothesized a lack of information and understanding, arguing for educational campaigns focusing on this population subgroup and offering educational materials and efforts in their native languages and by trusted representatives. This conclusion is in line with results from structured interviews addressing this point among refugees in the UK, the US, and Australia [1, 21, 22]. In these studies, concerns over vaccine content, side effects, lack of accessible information in an appropriate language, lack of trust in the health system, and low perceived need were found to be the main reasons for hesitance to vaccination [1, 21, 22]. Interstingly, the vaccination readiness in the cited countries was similar to that in Germany. Another explanation could be that refugees “adopt” skepticism against vaccination of the country of arrival. Further investigations are needed to compare vaccination readiness in countries with higher vaccination rates than in Germany. To us, it was interesting to see that one year later, over 90% of this population was vaccinated despite initial hesitation. It turned out that the vast majority were vaccinated during a campaign in the community shelters. In turn, it was unsurprising that doubts and reluctance against vaccination again characterized the new wave of (Ukrainian) refugees. This finding underlines that awareness-raising work and infection monitoring in common community shelters and among asylum seekers is not completed at a particular time point but is an ongoing task. In turn, our data prove the effectiveness of those efforts.

### Limitations

The results of our study must nevertheless be interpreted with caution, as the study has some limitations. Of note, our data cannot be extrapolated to draw any conclusions on the distribution of SARS-CoV-2 in refugees in Germany in general, as the study was limited to refugees living in community shelters. Still, our data align with cross-sectional and retrospective studies among people and similar circumstances in Australia, Germany, and France [11, 13, 14].

Although the study groups did not differ from the group of all asylum seekers in Lübeck concerning sex, age, and country of origin, we cannot exclude a selection bias, as participation was voluntary. Asylum seekers interested in the topic might have participated more likely and may thus be overrepresented. However, there also appears to be a bias in the Lübeck control group, as reflected by the willingness to be vaccinated in 2021 and the quote of the vaccinated subject one year later. The positive vaccination attitude in the control cohort was about 10% and 15% higher in the Lübeck control group than in the total population at the time of the survey, according to official information from the health authorities [23].

As an additional limitation, we could not analyze the genome of the RT-PCR-positive cases and cannot make any statement about virus variants. In February, at the second testing time point, the proportion of the B.1.1.7 was 15.4%, and of B.1.351 was 1.5% [24]; the dominating variant was the alpha variant. In May 2022, the dominating variants were BA.2.1 and BA.2.* [25].

The younger age of the asylum seekers and the predominantly male subjects in one of the refugee samples may bias the observed prevalence. However, the results in the control cohort did not differ significantly between males and females (AB- or PCR positive prevalence 3.1%, CI 2.2 to 4.1 females vs. 4.1%, CI 2.9 to 5.2 males) and were also in the young population (age 18–39) far lower than in the study population (3.8%, CI 2.5-5.0) [16]. Although there is a slight difference in sex distribution between both cohorts (57.9% vs. 44%) and a slightly lower mean age (37.7 vs. 45.6 years), this cannot account for the detected differences in prevalence. Interestingly, the lower ratio of infected people in the control cohort does not approach the higher frequency among refugees over time. It is possible that refugees suffered from infection just earlier. However, the infection frequency among refugees increases faster even after one year of the pandemic, according to our findings.

We cannot exclude that in some individuals, the antibody titer fell under the detection threshold after one year, although there is no evidence for that. Yet, as the frequency of an acute infection at TP1 and TP2 is increased among the refugees, a theoretical antibody decrease would lead to an underestimation of our results at TP3 and not influence the general conclusion of the study.

### Summary and conclusion

As we showed that SARS-CoV-2 transmission is already markedly increased among refugees living in high-income countries like Germany, the risk for most refugees in low-income countries must be expected to be even considerably higher [16]. The probability increases with each new refugee wave. Moreover, our results are in line with previous reports pointing to a higher infection risk for (homeless) people living in community shelters [11, 16], and recent research provided evidence for an association between infection risk and low socioeconomic status in general [21, 26]. Following the commitment to “leave no one behind” set by the World Health Organization, providing care for the most vulnerable groups, such as refugees, is mandatory for ethical reasons and to protect public health in general [27]. In conclusion, vaccination campaigns can be successful and, by that, help protect not only individuals but also larger populations. A further implication is that campaigns like those for vaccination for this particular subgroup have to be maintained as long as new migration movements reach the borders. In addition, our results are likely to be transferable to future pandemics of infectious diseases.

## Electronic supplementary material

Below is the link to the electronic supplementary material.

Supplementary Material 1

Supplementary Material 2

Supplementary Material 3

## Data Availability

The datasets generated and analyzed during the current study are not publicly available due to ethical concerns but are available from the corresponding author upon reasonable request.
