# Supplementary material for: Spreading of SARS-CoV-2 among adult asylum seekers in refugee community shelters in Lübeck, Germany between 2020 and 2022: a mixed-cohort observational and repeated cross-sectional study
Source: BMC Public Health. 2025 Apr 7;25:1301. doi: 10.1186/s12889-025-22120-9 (PMC11974058; doi:10.1186/s12889-025-22120-9)
Supplement: Supplementary file 1 — Supplementary Material 1 [file 12889_2025_22120_MOESM1_ESM.docx]

Supplemental material

*Further information about recruitment and study population*

To recruit asylum seekers, both the providers and the facility managers of each accommodation were contacted and personally informed about the study, and they all agreed to support the study. Information about the study was given to the facilities four weeks prior to the first testing time point. Interested participants were registered via the supervisors and managers at the facilities.
