# Supplementary material for: Spreading of SARS-CoV-2 among adult asylum seekers in refugee community shelters in Lübeck, Germany between 2020 and 2022: a mixed-cohort observational and repeated cross-sectional study
Source: BMC Public Health. 2025 Apr 7;25:1301. doi: 10.1186/s12889-025-22120-9 (PMC11974058; doi:10.1186/s12889-025-22120-9)
Supplement: Supplementary file 2 — Supplementary Material 2 [file 12889_2025_22120_MOESM2_ESM.docx]

Supplemental material

*Caregivers*

We also included ten caregivers from two homes at TP1 and TP2. No caregivers were included at TP3. All of the caregivers were vaccinated at that time-point due to legal restrictions. None of the caregivers participating in our study were PCR- or antibody positive at TP1 and TP2, respectively.
