# Supplementary material for: Spreading of SARS-CoV-2 among adult asylum seekers in refugee community shelters in Lübeck, Germany between 2020 and 2022: a mixed-cohort observational and repeated cross-sectional study
Source: BMC Public Health. 2025 Apr 7;25:1301. doi: 10.1186/s12889-025-22120-9 (PMC11974058; doi:10.1186/s12889-025-22120-9)
Supplement: Supplementary file 3 — Supplementary Material 3 [file 12889_2025_22120_MOESM3_ESM.docx]

Supplemental material

*Testing*

Infection detection and antibody testing were performed as previously described [12,14]. In brief, at TP1 and TP2, deep nasal and oropharyngeal swabs were performed by trained study personnel. Samples were combined into a single tube for each participant before RNA isolation. In addition, SARS-CoV-2 quantitative Real-Time PCR (RT-PCR) from dry swabs was performed at the National Reference Center for Mycobacteria at the Research Center Borstel (RCB), Leibniz Lung Center and the University Hospital Schleswig-Holstein (UKSH), Lübeck [12]. Nucleic acids were extracted, eluted, and quantitative RT-PCR was performed according to the manufacturer's instructions using the first WHO Emergency Use listed in In vitro diagnostics coronavirus (COVID-19) genesig® Real-Time PCR assay (Primerdesign™ Ltd), or the iTaq Universal Probes 1-Step kit (Bio-Rad, Feldkirchen) in conjunction with primers and probes from the SARS-CoV-2 (2019-nCoV) CDC qPCR Probe Assay (IDT, Leuven, Belgium).

Venous blood samples were used for antibody testing. The EUROIMMUN SARS-CoV-2 S1 IgG (#EI 2606-9601-2 G) ELISA was performed according to the manufacturer's instructions. Participants with an antibody titer against the S1 antigen of >1.1 (compared to an anti-S1 IgG-positive reference serum provided by the manufacturer in the ELISA detection system) were defined as anti-S1 IgG antibody-positive [12]. At TP3, there was no PCR testing, but only capillary blood samples were used for antibody testing only. Here, the EUROIMMUN SARS-CoV-2 NCP IgG (#EI 2606-9620-2 G) was used according to the manufacturer instructions.
